# Evaluation of a collaborative multi-disciplinary train-the-trainer program for first responders in chemical, radiologic and nuclear emergencies — a pre- and post-test

**DOI:** 10.1186/s12909-024-06024-7

**Published:** 2024-09-19

**Authors:** Pia Hedberg, Britt-Inger Saveman, Lina Gyllencreutz

**Affiliations:** Department of Nursing and Department of Diagnostic and Intervention, Center of Disaster Medicine, Umeå University, Umeå, Sweden

**Keywords:** CRN response, Care in hazardous environments, CiHE, Train-the-trainer programme

## Abstract

**Background:**

In significant events like chemical, biological, radiological, nuclear, and explosive (CBRNE) incidents, additional expertise in specific chemical substances becomes essential. Train-the-trainer programmes are used to increase knowledge and skills in a variety of fields and have been shown to be a cost-effective training method, eliminating the necessity of bringing in external experts or requiring participants to travel outside their region. Care in Hazardous Environments (CiHE) is one example of a course which comprises basic multi-disciplinary training together with personnel from rescue, police, and emergency medical services to prepare them to handle chemical and radioactive nuclear incidents. The train-the-trainer programme described in this study contains both theoretical and practical components, intended for instructors who will lead training on CiHE incidents. This study aimed to evaluate trainers’ level of knowledge before and after a train-the-trainer programme, as well as their thoughts about becoming an instructor i.e. the pedagogical competence for the Care in Hazardous Environments course.

**Methods:**

A pre- and post-test, along with an evaluation of open-ended response options were employed to assess the effectiveness of the train-the-trainer programme for teaching the basic course (CiHE). A total of 49 participants were enrolled in the programme.

**Results:**

Participants showed significant improvement in chemical, radiological and nuclear (CRN) response knowledge in two of the eight questions between the pre- and post-tests. The two questions that improved pertained to chemical substances and basic principles of radiation protection. Instructors trained in the train-the-trainer programme are intended to bring new knowledge, incorporate a rarely discussed topic into instruction regularly, and de-stigmatise CRN incidents by helping raise the minimum competency levels in their respective organisation.

**Conclusion:**

An effective response to CBRNE events begins with readiness. First responders must be prepared and possess knowledge of both CRN components as well as protective gear to keep themselves and others safe at the incident scene. This study shows the importance of the train-the-trainer programme in continuing to educate police, and personnel from rescue and emergency medical services in CiHE, enable them to collaboratively prepare to handle CRN incidents.

## Background

The escalation of modern technological advancements and the shifting landscape of global security threats have contributed to the global growth of chemical, biological, radiological, nuclear, and explosive (CBRNE) incidents. In this context, it is crucial for governments and organisations worldwide to develop effective strategies for preparedness and response in the case of such incidents, considering their potential for widespread devastation and significant impact on public health and safety. Therefore, emergency response teams and healthcare professionals need to be well-equipped and adequately trained to handle CBRNE incidents, given their potential catastrophic consequences and the intricacies involved in managing these events [1, 2]. Studies conducted in European hospitals have highlighted a deficiency in education and preparedness for CBRNE incidents [3, 4]. Similarly, research has revealed that emergency medical service (EMS) personnel often lack sufficient preparedness to respond to disasters like CBRNE incidents [5–7]. EMS personnel have expressed feeling unsafe during CBRNE events, with uncertainty regarding the use of personal protective gear [8, 9].

CBRNE incidents present a complex set of challenges. Research has shown [10] that first responders, including police, rescue services, and EMS personnel, require a comprehensive understanding of hazardous materials and the associated precautions that should be taken during a CBRNE incident. Without this readiness, there is a risk of being unable to identify chemical agents based on patient complaints and symptoms, thereby hindering the initiation of disaster response procedures. Moreover, there is an increased risk of secondary contamination among healthcare workers and in the surrounding environment, potentially resulting in the shutdown of medical units [11]. Additionally, healthcare workers may harbour concerns about the physical and mental harm associated with potential exposure to toxic substances, e.g., radioactive, nuclear, or chemical materials. Research has emphasised that when healthcare professionals lack adequate preparedness, only 30% of them are willing to support disaster response efforts [12].

To enhance knowledge and competence in handling CBRNE incidents, it is essential to create educational curricula for healthcare personnel [13]. Training programmes that encompass both theoretical and practical components have proven valuable in establishing enduring CBRNE preparedness among EMS personnel [14]. Effective training methodologies for managing mass casualties in CBRNE incidents, such as simulations and table-top drills [15, 16], are of paramount importance [17]. Research indicates that a combination of theoretical and practical training enhances the knowledge and proficiency of EMS personnel in responding to CBRNE incidents [18, 19].

Train-the-trainer programmes are a type of education aimed at training trainers. This type of programme is used in a wide variety of fields for workforce development, including public health preparedness [20], such as occupational safety [21], and in clinical interventions [22]. Trainers are key players in knowledge translation by teaching trainees, for example other healthcare professionals to improve their knowledge, skills, and attitudes [23]. The existing literature points out that there is not yet sufficient evidence to conclude whether the train-the-trainer programme is more effective compared to other programmes [24]. Key to obtaining consistent success with training programmes is having a systematic approach to measurement and evaluation [25]. Evaluation in the training context involves gathering information on the impacts of a training programmes and then appraising the worth of the training based on said information. Train-the-trainer programmes must be evaluated with repeated measures to better support trainers’ competencies development [26–28]. It is important to consider trainers, as they are primarily responsible for enhancing the required theoretical knowledge and practical skills in an efficient train-the-trainer course. To bridge the gap in comprehensive research on the effectiveness of train-the-trainer programmes [28], one approach is to measure the learning outcomes of trainers. This approach can offer valuable insights into effective methods of supporting trainers and documenting the efficacy of the train-the-trainer programme.

This study aimed to evaluate trainers’ level of knowledge before and after a train-the-trainer programme, as well as their thoughts about becoming an instructor, i.e. the pedagogical competence for the Care in Hazardous Environments course.

## Methods

### Study design

Meant for the background a systematic review of existing literature was conducted by the first author.

In this study, a pre- and post-test, along with an evaluation with open-ended response options, were employed to assess the effectiveness of the train-the-trainer programme for teaching the basic course Care in Hazardous Environments (CiHE). The purpose of the train-the-trainer programme was to educate instructors and enhance their knowledge of CRN response, equipping them with pedagogical tools to further educate personnel in EMS, police, and rescue services.

Eligibility for the train-the-trainer programme required participants to have completed the CiHE course and hold either a vocational education or a university degree in their field. Additionally, they were required to have significant practical experience, ensuring they are well-prepared to deliver high-quality training. In the following sections, CiHE course content is described first, followed by components specific to the train-the-trainer programme.

The development of the CiHE and the train-the-trainer programme involved experts from various Swedish organizations, including The National Board of Health and Welfare, The Swedish Defence Research Agency, The Police Authority, and The Swedish Civil Contingencies Agency. This collaborative effort ensures that both the CiHE course and the train-the-trainer programme are standardized and applicable nationwide.

### The CiHE course

The CiHE course consists of basic and applied training. The basic training provides theoretical knowledge and tactics to personnel involved in the early rescue phase of CRN incidents for effective multi-disciplinary collaboration with affected individuals. This simultaneously offers insights into a safer work environment for personnel and more efficient rescue operations. The training is primarily targeted at high-risk environments where CRN substances are presumed to be involved, including fire and smoke. However, the tactics and approach should be applicable even in other high-risk environments.

The target audience is the first responders who may be first on the scene or arrive during the early stages of an incident, with a focus on working closely with affected individuals. The theoretical part consists of nine chapters of self-directed e-learning material. Each chapter covers a specific topic, such as protective gear. There is a mix of text and films, and at the end of the chapter, there are multiple choice review questions. The theoretical part is followed by applied training, practicing hands-on skills in multidisciplinary teams, led by instructors to apply and reinforce the trainers’ knowledge. Completion of the CiHE course is a requirement to attend the train-the trainer programme.

### Train-the-trainer programme

The development of the train-the-trainer programme as well as the CiHE course involved experts from various Swedish organizations, including The National Board of Health and Welfare, The Swedish Defence Research Agency, The Police Authority, and The Swedish Civil Contingencies Agency. This collaborative effort ensures that both the CiHE course and the train-the-trainer programme are standardized and applicable nationwide. Previous research indicates that a train-the-trainer programme could include interactive components such as lectures, practice, live cases, and role play [11, 29].

The train-the-trainer programme includes both theoretical and practical components over the course of three days. During the first two days, theoretical lectures were intertwined with practical exercises, such as the use of protective gear, decontamination, and practical labs concerning CRN incidents. The theoretical content was delivered by experts in CRN, emergency care, and a special pedagogical expert for the pedagogical learning lecture. All the practical exercises and labs were evaluated with oral feedback from the teachers at the end of the days.

**Fig. 1:**
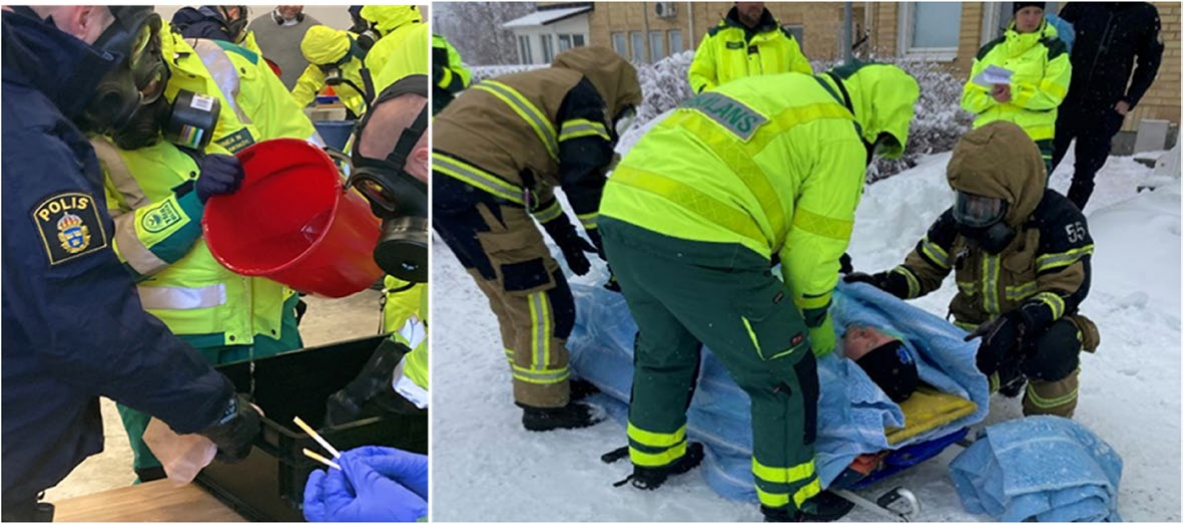
Example of case scenarios

On the third day, three case scenarios involving CRN incidents were introduced. The trainers assumed various roles, acting as both instructors and participants. During the preparation of these scenarios, trainers had access to CRN experts, whom they could consult with any questions regarding specific cases. Following the case scenarios, trainers received feedback on their teaching skills from experts, teachers and peers (Table 1, Fig. 1).

**Table 1:** Train-the-trainer programme schedule with theoretical lectures (TL) and practical exercises (PE)

Day 1
Introduction (10 min)
Baseline Measurements (20 min)
Overview of CiHE (30 min) (TL)
Basic Chemistry and Resources in Sweden (15 min) (TL)
Gases and Combustibles (40 min) (TL)
Chemical Medicine and Chemical Weapons Attack (60 min) (TL)
Pedagogical Lecture (60 min)
Putting on and Taking Off Protective Gear & Movement Techniques (30 min) (PE)
Acid and Base Lecture and Lab (60 min) (TL, PE)
Instructor Group Case Preparation (90 min) (PE)
**Day 2**
Review of Day 1 (15 min)
Basic Radiation Physics (60 min) (TL)
Quantities and Units, Exposure and Exposure pathways, Radiation Protection Rules (45 min) (TL)
Reference Levels and Zones (15 min) (PE)
Radiologic and Nuclear Medical Lecture and Medical Effects (60 min) (TL)
Radiation Measurement, Control Measurement, Laws, and Regulations for Nuclear Weapons (60 min) (TL)
Personal Decontamination Exercise (CRN), Life-Saving Personal Decontamination at the Scene (90 min) (PE)
Group Presentation of Basic CASE (90 min) (PE)
**Day 3**
Review of Day 2 (15 min)
Group Presentation of Basic CASE (45 min) (PE)
Auto-injector Instruction and Practice (30 min) (TL, PE)
Workshop on the Instructor Role (65 min) (TL)
Discussion on Practical Application of Learned Material (45 min) (TL)
Website and Mentimeter License Overview (15 min) (TL)
Course Closure (15 min)

### Study population

A total of 49 people (11 women and 38 men) participated in the train-the-trainer programme during the three sessions held during fall 2022 and spring 2023. Fifteen participants attended the first programme in southern Sweden, 17 participants attended the programme in central Sweden, and 19 participants attended the programme in northern Sweden. The participants represented all three organisations, EMS, police, and rescue service. In addition to the CiHE course, 34 participants had previous education in CBRNE, and 35 participants had experience using C-protective equipment (Table 2).

**Table 2:** Background characteristics

Age	Female	Male	Total
20–29 year	1	5	6
30–39 year	7	19	26
40–49 year	3	9	12
50–59 year	0	5	5
**Total**	**11 (22%)**	**38 (78%)**	**49 (100%)**
**Occupational category**			
EMS personel^a^	9	19	28
Physician	1	0	1
Rescue service^b^	1	11	12
Police	0	8	8
**Total**	**11 (22%)**	**38 (78%)**	**49 (100%)**

^a^ Ambulance nurses and nurses

^b^ Fire engineers, firefighters, and team leaders

### Data collection tools

The pre- and post-tests were conducted using a questionnaire that was sent to all participants before and after their participation in the train-the-trainer programme. The pre-test questionnaire also included background questions, such as age, employment, and previous education in CBRNE events. The post-test questionnaire was sent to participants one week after they had completed the train-the-trainer programme.

The questionnaire used in both the pre and post-test consisted of eight review questions covering the theoretical content of the CiHE course. The questions were multiple-choice, with only one correct answer. The same questions were used in both the pre and post-test. The questions were as follows: What are examples of chemical substances? What are examples of RN events? What are examples of warning signs of potential events involving hazardous substances? What is the wind direction if there is a south-easterly wind? When is a risk assessment carried out? Which of the options is implemented *first* in the event of an incident involving dangerous substances? Why is it important to know the properties of hazardous substances? What are the three basic principles of radiation protection?

**Open-ended questions** At the end of the programme, four open-ended questions were distributed to the participants: What characteristics are important for an instructor? What are my strengths? What challenges will I face? What are my expectations in the role of an instructor? All participants (*n* = 49) reflected on these questions and recorded their answers on paper without including their names before leaving the programme. Consequently, the collected responses were anonymous to the authors, and no identifiers were assigned to the participants.

### Data analysis

Analysis was conducted using IBM SPSS statistics, version 28. Descriptive statistics are presented at both the individual and group level. Group comparisons for the pre- and post-test were made using Fisher’s exact test due to the small sample size. Significant differences between the pre- and post-tests were identified when the p value was less than 0.05.

A qualitative thematic analysis of the responses to the open-ended questions was also conducted. Thematic analysis involves identifying, analysing, and interpreting patterns of meaning, known as themes, within qualitative data. This method uncovers patterns in participants' lived experiences, viewpoints, perspectives, behaviours, and practices, both within and across individual datasets [30]. The first author reviewed all responses, sorted and labelled them as codes. The codes were then discussed among all the authors and abstracted into sub-themes and one main theme. Quotations from participants are presented in the results.

### Ethics statement

This study was performed according to the Helsinki Declaration [31]. Study participants are professionals and not patients, thereby not regulated by Swedish Code of Statutes [32]. All participation in the study was voluntary. Full informed consent was given by participants with the option to withdraw from the study at any time. Results were presented at the group level, and individual participants cannot be identified.

## Results

The results include findings from the pre- and post-tests as well as the themes related to the role of being an instructor, which were gathered from the open-ended questions.

### Pre- and post-tests

The answers from the pre and post-tests are presented in Table 3. The results showed an increase in knowledge between the pre- and post-tests in two areas: examples of chemical substances and basic principles of radiation protection. Concerning the remaining questions, there were no significant differences.

**Table 3:** Correct answers from the pre and post-tests

	Pre-test (*n* = 49) Correct answer (%)	Post-test (*n* = 23) Correct answer (%)	*p*-value
Chemical (C) substances	22 (45)	19 (83)	*p* = 0.004^*^
Radiologic and Nuclear events	46 (98)	21 (91)	*p* = 0.652
Wind direction	41 (87)	21 (91)	*p* = 0.485
Warning signs	39 (80)	22 (96)	*p* = 0.092
Risk assessment.	47 (96)	23 (100)	*p* = 1
Hazardous substances?	45 (96)	22 (96)	*p* = 1
Properties of hazardous substances?	34 (70)	15 (66)	*p* = 0.790
Principles of radiation protection?	35 (71)	22 (96)	*p* = 0.027^*^

^*^Statistical significance

### Thematic result

The qualitative analysis revealed one main theme—being an instructor—supported by three sub themes: characteristics, strengths, and challenges.

### Characteristics of an instructor

Participants described the importance of being a role model. This include listening to everyone's opinions, being well-prepared and pedagogical, keeping lectures interesting, involving participants, and finding a suitable balance between trainers and trainees. Moreover, being socially competent and setting a reasonable level of expectation for the target audience were identified as important characteristics.

Being a lively trainer who finds interest in the subject and includes participants in an inspiring way.

Participants also described that the train-the-trainer programme provided them with insights into how to be inclusive and open-minded, and the importance of allowing time for trainees to reflect and speak without interruption.

### Personal strengths

Participants expressed various strengths, such as being clear communicators and confident leaders. They also valued the ability to create a relaxed atmosphere, demonstrate humility, understand group dynamics, being encouraging and think creatively.

Enjoying meeting new people, and sharing knowledge and experiences, and having genuine interest in the subject.

In addition, having structured and clear communication and the ability to adapt, having great planning skills, and being good at explaining concepts were described as necessary by participants.

### Challenges as an instructor

Participants described several challenges associated with being an instructor These included creating interest and understanding for rare incidents, de-stigmatising the subject while maintaining respect, making practical exercises as realistic as possible, allocating roles during exercises, and avoiding technical issues. Computer-based disruptions were also seen as a challenge during exercises. Moreover, dealing with sceptical, uncertain, and critical participants was also considered challenging. However, participants described that the programme had provided them with useful tools to manage challenging situations.

It will be a challenge to capture participants' interest, considering that such incidents are rare and seldom events.

Participants also expressed concerns at the organisation level, particularly regarding the availability of sufficient time from their employers for planning and execution. Collaboration with fire and police departments was also seen as a challenge due to the involvement of three different organisations.

Following the train-the-trainer programme, participants expected to bring knowledge of a rarely discussed topic on a regular basis and to help de-stigmatise CRN incidents by raising the minimum competence levels within their respective organisation. They emphasised the importance of facilitating effective cooperation and knowledge exchange between EMS, police, and rescue services.

## Discussion

The main result of this study is that participants in the train-the-trainer programme demonstrate a significant increase between pre- and post-tests in two of eight questions and in the main theme: being an instructor.

The pre- and post-tests showed improved knowledge in the questions of chemical substances and radiation protection. However, no significant differences were found in the six remaining questions. This may be explained by how the questions were addressed or because participants’ knowledge levels were already high in the pre-test. Another study showed that the participants increased their knowledge, which led to them feeling safer when responding to CRN incidents [7]. These findings correspond with previous research, which suggest that the train-the-trainer programme has possibilities to be an effective method to prepare first responders [28]. Our train-the-trainer programme is well-designed and aligns with the basic course CiHE. This is comparable to earlier research indicating that well-designed train-the-trainer programmes, with a comprehensive integration of various interactive components, effectively prepare trainers to teach first responders how to manage CRN incidents.

The thematic analysis revealed that the train-the-trainer programme provided participants with useful tools, knowledge, and resources for delivering high-quality training. However, the results also show challenges at an organisational level, particularly in finding time to collaborate among the three different organisations.

The train-the-trainer programme plays a critical role in enhancing preparedness for CRN incidents. It has been reported [29] that first responders (police, firefighters, and EMS personnel) should know and be able to identify hazardous materials they may encounter in any CBRNE incident. They should also have knowledge of the precautions to take against adverse situations related to exposure to dangerous substances, understand post-exposure symptoms and findings, and know how to respond. Training for these situations should be practical and contribute to the development of the team’s intervention skills [29, 33, 34]. Based on our findings, our train-the-trainer programme fulfils these requirements.

Our findings indicate numerous advantages and insights associated with a train-the-trainer programme. These include the benefit of utilising local trainers who possess a deeper understanding of contextual issues, allowing for customised training. It also encourages increased collaboration among organisations, fostering practical experience and networks development. Additionally, a train-the-trainer programme has proven to be a cost-effective training method, eliminating the need for external trainers or requiring participants to travel outside their region [20, 21]. While challenges may arise in train-the-trainer programmes, future instructors can overcome these challenges through innovation, collaboration, and a commitment to continuous improvement [23, 26]. By addressing these challenges, future instructors can ensure that individuals and organisations are better prepared to respond to CRN incidents and safeguard public safety.

###  Strengths and limitations of this study

The structure of the train-the-trainer programme, which spans three days, allows for a balanced blend of theoretical knowledge and practical application. The involvement of experts in CRN incidents in designing the theoretical content and delivering lectures enhances the programme's credibility and relevance. Practical exercises involving the use of protective gear and decontamination procedures, as well as hands-on lab sessions focusing on CRN incidents, provide participants with valuable experience. The inclusion of case scenarios at the end of the programme further enriches the learning experience. These scenarios not only test participants' understanding of CRN incidents but also their pedagogical skills. The active involvement of trainers in these scenarios, assuming dual roles as both instructors and performers, likely facilitated a deeper understanding of the material.

A total of 49 participants who answered the pre-test, while fewer than half of that number completed the post-test. The missing data may have affected the results. However, the small sample from the post-test showed significant differences from the pre-test in the questions pertaining to chemical substances and the basic principles of radiation protection. Boyd et al. [35] described many characteristics that may influence the outcome of a train-the-trainer programme, such as enthusiasm, attractiveness, expertise, and trustworthiness.

The participants who completed the post-test may have been those with a great enthusiasm for the content and the programme. When asked about chemical substances and principles of radiation protection, the results showed a significant increase, but all other responses showed no significant differences between the pre- and post-test. Caution must be taken, as the absolute figures could mean that those who knew the answers in the first round also participated in the second round. Those who did not know the answers may not have participated in the post-test.

The first author attended all three train-the-trainer programmes. On the last day of the course, the first author distributed a paper with four open-ended questions, which all participants answered. The results are strengthened by the fact that the responses from the open-ended questions were analysed and discussed within the research group to reach a consensus on the theme and sub-themes.

## Conclusions

CBRNE events seldom occur, yet first responders, that is personnel from the rescue services, police, and EMS, must be ready to act as a multidisciplinary team. An effective response to CBRNE events starts with readiness. First responders must be prepared and possess knowledge about, for example CRN components and protective gear, to act in a safely at the incident scene. This study shows the importance of a train-the-trainer programme in continuing to educate personnel from police, rescue services and EMS, enable them to become instructors for the basic CiHE course. This ensures that as many first responders as possible can be prepared to handle CRN incidents. Both the CiHE course and the train-the-trainer programme ought to be applicable nationwide.

## Data Availability

Data generated from this study are available from the corresponding author upon reasonable request.

## Data availability

Data generated from this study are available from the corresponding author upon reasonable request.
